# Diagnostic and economic evaluation of new biomarkers for Alzheimer’s disease: the research protocol of a prospective cohort study

**DOI:** 10.1186/1471-2377-12-72

**Published:** 2012-08-10

**Authors:** Ron LH Handels, Pauline Aalten, Claire AG Wolfs, Marcel OldeRikkert, Philip Scheltens, Pieter Jelle Visser, Manuela A Joore, Johan L Severens, Frans RJ Verhey

**Affiliations:** 1 Alzheimer Centre Limburg, School for Mental Health and Neuroscience (MHeNS), University Medical Centre, P.O. Box 5800, 6202, AZ, Maastricht, the Netherlands; 2 Alzheimer Centre Nijmegen, Radboud University Nijmegen Medical Centre, Nijmegen, the Netherlands; 3 Alzheimer Centre, Department of Neurology, VU University Medical Centre, Amsterdam, the Netherlands; 4 Maastricht University, CAPHRI School for Public Health and Primary Care, Faculty of Health Medicine and Life Sciences, Department of Health Organization, Policy and Economics, P.O. Box 616, 6200, MD, Maastricht, the Netherlands; 5 Institute of Health Policy and Management, Erasmus University Rotterdam, P.O. Box 1738, 3000, DR, Rotterdam, The Netherlands; 6 Department of Psychiatry and Neuropsychology, University Hospital of Maastricht/Alzheimer Centre Limburg, P.O. Box 5800, 6202, AZ, Maastricht, The Netherlands

## Abstract

**Background:**

New research criteria for the diagnosis of Alzheimer’s disease (AD) have recently been developed to enable an early diagnosis of AD pathophysiology by relying on emerging biomarkers. To enable efficient allocation of health care resources, evidence is needed to support decision makers on the adoption of emerging biomarkers in clinical practice. The research goals are to 1) assess the diagnostic test accuracy of current clinical diagnostic work-up and emerging biomarkers in MRI, PET and CSF, 2) perform a cost-consequence analysis and 3) assess long-term cost-effectiveness by an economic model.

**Methods/design:**

In a cohort design 241 consecutive patients suspected of having a primary neurodegenerative disease are approached in four academic memory clinics and followed for two years. Clinical data and data on quality of life, costs and emerging biomarkers are gathered.

Diagnostic test accuracy is determined by relating the clinical practice and new research criteria diagnoses to a reference diagnosis. The clinical practice diagnosis at baseline is reflected by a consensus procedure among experts using clinical information only (no biomarkers). The diagnosis based on the new research criteria is reflected by decision rules that combine clinical and biomarker information. The reference diagnosis is determined by a consensus procedure among experts based on clinical information on the course of symptoms over a two-year time period.

A decision analytic model is built combining available evidence from different resources among which (accuracy) results from the study, literature and expert opinion to assess long-term cost-effectiveness of the emerging biomarkers.

**Discussion:**

Several other multi-centre trials study the relative value of new biomarkers for early evaluation of AD and related disorders. The uniqueness of this study is the assessment of resource utilization and quality of life to enable an economic evaluation. The study results are generalizable to a population of patients who are referred to a memory clinic due to their memory problems.

**Trial registration:**

NCT01450891

## Background

Alzheimer’s disease (AD) and other dementing disorders are common in the elderly, with a worldwide prevalence estimated in 2010 at 35.6 million, which will double every 20 years to 115.4 million in 2050. AD has a substantial impact on the person who suffers from the disease, his or her family and society [1]. AD affects a person’s cognition, behavior and functional ability, and it is one of the leading causes of disability in older people living in developed countries [2].

The NINCDS-ADRDA criteria [3] are currently applied in diagnostic guidelines [4,5] to determine AD aetiology. Scientific knowledge, advanced imaging techniques and cerebrospinal fluid analyses have evolved since the publication of these criteria in 1984. This has led to much debate and the proposition of new clinical and research criteria to enhance diagnostic accuracy, even at the stage of early clinical symptoms [6-10]. These criteria distinguish between the AD pathophysiological process and the clinically observable syndrome to enable determination of AD in a pre-dementia state; e.g. mild cognitive impairment (MCI). In the end the criteria are meant to support therapy decision making (when effective treatments are available) or to determine the likelihood of cognitive and functional progression to a more severe disease state. Emerging biomarkers are attributed a more prominent role in the diagnostic criteria; amyloid β42, total tau and phosphorylated-tau in the cerebrospinal fluid (CSF), amyloid tracer uptake and fluorodeoxyglucose (FDG) in positron emission tomography (PET), hippocampal volume and medial temporal atrophy in structural magnetic resonance imaging (MRI) and single photon emission tomography (SPECT) perfusion imaging. However, validation of the criteria is needed before adoption of the proposed role of new biomarkers in clinical practice [9].

The ultimate goal of diagnostic testing is to guide disease management in order to improve patient outcomes and patient well-being. Tests that lack this potential should be regarded obsolete [11,12]. This has raised an urgent need for health technology assessment to address the direct, intended consequences of technologies as well as the indirect, unintended consequences for the evaluation of the value of diagnostic strategies including biomarker for AD compared to current clinical practice. Evidence is needed to support decision makers on the adoption of new diagnostic tests in clinical practice to enable efficient allocation of health care resources.

## Study aim

The general aim of the study is to assess the clinical and economic value of current, emerging and novel (to be developed) techniques for an early diagnosis of AD and related disorders. In this paper the methodology is described.

The research goals are:

1. To assess the diagnostic test accuracy of the current clinical standard diagnostic work-up and emerging diagnostic biomarkers in MRI, PET and CSF

2. To assess costs and effects for the follow-up period to perform a cost-consequence and cost-effectiveness analysis

3. To develop a preliminary economic model to assess the uncertainty surrounding long-term cost-effectiveness of diagnostic strategies

## Methods/design

### Study design

A cohort design was chosen because an assessment of test combinations within a randomized controlled trial would require the evaluation of many diagnostic strategies for which the number of subjects needed would exponentially increase [13]. To determine the diagnostic value of emerging biomarkers for AD and related disorders both a clinical diagnosis and diagnosis based on emerging biomarkers (index tests) are compared with a reference diagnosis. Due to limited ability of biopsy (which is unethical) or autopsy (which requires follow up until death) a two-year follow up of the clinical course is used as a proxy to obtain information on the state of the disease at baseline; so-called delayed-type cross-sectional accuracy study design [12]. Four academic memory clinics (Leiden University Medical Centre, Maastricht University Medical Centre, Radboud University Nijmegen Medical Centre and VU University Medical Centre) specialized in the diagnosis and treatment of memory disorders participate in the study. Two memory clinics are settled within a department of geriatrics, one within neurology and one within psychiatry. The study is performed within the framework of CTMM, the Center for Translational Molecular Medicine (http://www.ctmm.nl), a Dutch public-private partnership; project LeARN (grant 02 N-101).

### Subjects

For the study, 241 consecutive patients of the participating memory clinics who were suspected of having a primary neurodegenerative disease were included for participating in the study from October 2009 to May 2011; this included all patients with subjective and/or objective memory complaints. Eligibility criteria were chosen to represent the current clinical situation and enable generalisability to clinical practice (see Table 1). Informed consent was obtained from both the patient and the informal caregiver. Gender, age and reason for refusal were obtained for patients unwilling to participate.

**Table 1:** Eligibility criteria for subject selection

*Inclusion criteria*	
-	All new consecutive patients of the participating memory clinics who are suspected of having a primary neurodegenerative disease
-	Mini-Mental State Examination: ≥ 20
-	Clinical Dementia Rating: 0 – 1
-	Availability of a reliable informer or proxy (who visits or contacts the patient at least once a week)
*Exclusion criteria*	
-	Normal Pressure Hydrocephalus, Huntington’s disease
-	Less than two years ago a transient ischaemic attack (TIA) or cerebral vascular accident (CVA) or TIA/CVA followed (within three months) by cognitive impairment
-	Psychiatric history (schizophrenia, bipolar disorder or psychotic problems (not otherwise specified), less than 12 months ago)
-	Major Depression according to the DSM-IV, less than 12 months ago
-	Cognitive problems due to excessive alcohol use (based on clinical judgement)
-	Brain tumour, epilepsy, encephalitis
-	Probably not available for follow-up

### Data assessment

Each centre collects a minimum dataset of clinical information based upon the dataset protocol used for The String of Pearls Initiative – Pearl Neurodegenerative Diseases (http://www.string-of-pearls.org), cost data and data on emerging biomarkers. Table 2 provides an overview of all patient and informal caregiver assessments. Assessments take place at baseline and at 12 and 24 months follow up during a visit (from both patient and informal caregiver) to the memory clinic. Furthermore, several questionnaires were composed in a booklet to measure resource consumption and quality of life. This is filled out by the informal caregiver at baseline, 3, 12 and 24 months.

**Table 2:** Overview of patient and informal caregiver assessments at baseline and follow-up

**Outcome measure**	**Operationalization/type of instrument**	**B**	**T3**	**T12**	**T24**
***Clinical data***					
Demographic data	History taking	P		P	P
Cognitive impairment	Mini-mental State Examination	P		P	P
Dementia severity	Clinical Dementia Rating	P		P	P
Functional disability	Disability Assessment for Dementia	P		P	P
Neurological and physical examination	Neurological assessment and evaluation of co-morbidities	P		P	P
Neuropsychiatric problems	Neuropsychiatric Inventory	P		P	P
Depression	Geriatric Depression Scale 15	P		P	P
Cerebral atrophy and white matter lesions	Structural MRI (T1-weighted, T2-weighted and FLAIR)	P			
Neuropsychological Assessment	Rey’s Verbal Learning Test, Visual Association Test, Digit-Span, Letter Digit Substitution Test, Stroop Color-Word Test, Trail Making Test	P		P	P
***Quality of life data***					
Patient generic quality of life	EQ-5D	I*/P	I*	I*/P	I*/P
Patient disease specific quality of life*	QoL-AD	I *	I *	I *	I *
Caregiver generic quality of life*	EQ-5D	I	I	I	I
Caregiver disease specific quality of life*	QoL-AD	I	I	I	I
Caregiver burden*	Sense Of Competence	I	I	I	I
Care-related quality of life*	Carer Quality of Life	I	I	I	I
Cost data*					
Resource utilization and caregiver time	RUD-Lite	I	I	I	I
Work productivity and absence	Productivity and Disease Questionnaire	I	I	I	I
Consequences of informal caregiving on paid or unpaid work	Health and Labour Questionnaire	I	I	I	I
Other resource use		I	I	I	I
***Emerging biomarker data***					
Functional connectivity	Resting state functional MRI	P			
White matter integrity	Diffusion tensor imaging	P			
Hippocampal volume	Structural MRI	P			
Glucose metabolism	Fluorodeoxyglucose PET†	P			
Amyloid plaque deposition	Pittsburgh compound B PET†, CSF Aβ1-42	P			
Tau	CSF total tau, CSF phosphorylated tau	P			

*These items were assessed by the informal caregiver by means of a booklet in which several questionnaires were composed for resource consumption and quality of life.

†Data on this item are only collected at the memory clinic of the VU University Medical Centre Amsterdam.

Abbreviations: B, Baseline; FLAIR, Fluid attenuation inversion recovery; I, Information retrieved from informal caregiver; MRI, Magnetic Resonance Imaging; P, Information retrieved from patient; PET, positron emission tomography; QoL-AD, Quality of Life Alzheimer’s Disease scale; RUD-Lite, Resource Utilization In Dementia; T3, 3 month follow-up measurement; T12, 12 month follow-up measurement; T24, 24 month follow-up measurement.

#### Clinical data

Demographic and medical information is retrieved from an open interview with both patient and informal caregiver and physical examination by a clinician.

The Mini Mental State Examination is used to detect cognitive impairment, to assess its severity and to monitor cognitive changes over time [14]. The Clinical Dementia Rating scale (CDR) [15,16] provides a global rating of dementia severity. The Geriatric Depression Scale-15 [17] is applied to detect depression. Patient’s behavioural and psychological problems are measured by the Neuropsychiatric Inventory (NPI) [18]. The Disability assessment for Dementia (DAD) is assessed to evaluate basic and instrumental activities in daily activities [19]. The information of both the NPI and DAD is obtained from a caregiver familiar with the patient’s behaviour by means of a semi structured interview. Caregiver’s burden of care is assessed using the disease specific Sense of Competence Questionnaire (SoCQ) [20,21].

Neuroimaging markers include medial temporal lobe atrophy measurements and white matter lesions which are qualitatively scored based on 3 T MRI scan images.

Neuropsychological examination consists of a standardized battery of cognitive tests performed by a (neuro)psychologist. Tests include Rey’s Verbal Learning Test [22,23], Visual Association Test [24], and Digit-Span [25] to assess memory; Letter Digit Substitution Test [26] to assess mental processing rate; and Stroop Color-Word Test [27] and Trail Making Test [28,29] to assess attention, concentration and interference. Raw scores were converted to z-scores, adjusting for age, education and gender.

#### Quality of life data

Patient’s generic quality of life is measured by the EQ-5D instrument. It was developed and validated in a number of European countries including the Netherlands [30-32] and it has been validated in patients with dementia [33,34]. The EQ-5D describes health status according to five three-level dimensions, which yields 243 potential combinations of health states. Each combination leads to a utility score by means of an additive function derived from the UK general population [35,36].

Patient disease specific quality of life is measured by the validated Quality of Life – Alzheimer’s Disease scale (QoL–AD) [37,38]. It has 13 items covering the domains of physical health, energy, mood, living situation, memory, family, marriage, friends, self as a whole, ability to do chores around the house, ability to do things for fun, money, and life as a whole. Scale scores range from 13 to 52 with higher scores indicating greater QoL. An improvement of 3 points on the QoL-AD is judged as clinically relevant as this indicates a change of well-being on one of the domains from very poor to excellent [39].

Patient EQ-5D is assessed by the patient during the visit to the memory clinic. Furthermore, the informal caregiver judges the EQ-5D and QoL-AD for the situation of the patient and for his/her own situation and fills this out in the booklet of questionnaires.

Care-related quality of life of informal caregivers is assessed by the CarerQol[40]. It combines seven important burden dimensions with a valuation component (a visual analogue scale (VAS)) for happiness. The seven burden dimensions are 1) fulfilment; 2) relational problems; 3) mental problems; 4) problems with daily activities; 5) financial problems; 6) support; and 7) physical problems. The CarerQol-VAS ranges from 0 (“completely unhappy”) to 100 (“completely happy”) and has been validated in a Dutch sample of heterogeneous caregivers.

#### Cost data

Cost data are retrieved by the composed booklet of questionnaires. Patient resource utilization and caregiver time, which often contains productivity losses, are assessed by means of the short version of the Resource Utilization in Dementia-questionnaire (RUD-lite). This instrument has been validated and proved to register over 95% of the costs involved in AD-care [41]. Work status, income, and productivity losses of both the patient and caregiver are assessed by the adjusted PRODISQ (PROductivity and DISease Questionnaire) [42]. The consequences of informal caregiving on paid or unpaid work are assessed by the Health and Labour Questionnaire on a two-week scale on which is indicated whether one was ill, ill by caregiving or not ill [43]. Additional questions are asked referring to the number of visits to various health care professionals, resources or aids that are bought and other out-of-pocket costs.

#### Emerging biomarker data

Biomarkers can be divided into two categories, one reflecting the presence of beta-amyloid protein (Aβ) and one reflecting neuronal degeneration or injury. A pathological cascade is hypothesized in which basically Aβ markers become abnormal first, followed by neuronal injury [9,44].

The biomarkers included in this project are outlined in Table 3. CSF is collected and Amyloid β42, total tau and phosphorylated-tau are analyzed using a standardized quantitative method. FDG uptake and Pittsburgh compound B binding (PiB) on PET are both qualitatively rated by a radiologist and quantitatively analyzed by standardized methods. Whole brain and hippocampal volume, white matter integrity and functional connectivity derived by MR imaging are quantitatively analyzed by a researcher. These tests are not part of the current routine clinical diagnostic procedure. They are judged and analyzed independently and blindly. The outcome of each test is dichotomous or continuous, both for AD aetiology and progression of cognitive decline.

**Table 3:** Included biomarkers in the project categorized by reflecting Aβ or neuronal injury

**Technique**	**Aβ accumulation**	**Neuronal dysfunction**
CSF	Amyloid β42 in the CSF	total tau and phosphorylated-tau in CSF
PET	amyloid tracer uptake in PET	fluorodeoxyglucose PET
MRI		White matter integrity (DTI)
		Functional connectivity (rsfMRI)
		Whole brain volume
		Hippocampal volume

Abbreviations: Aβ, beta-amyloid protein; CSF, cerebrospinal fluid; DTI, diffusion tensor imaging; MRI, magnetic resonance imaging; PET, positron emission tomography; rsfMRI, resting state functional magnetic resonance imaging.

### Baseline clinical diagnosis and reference diagnosis

Current clinical practice diagnosis is reflected by a consensus procedure among experts using baseline clinical information only, excluding any information of emerging biomarkers to prevent underestimating their accuracy.

The reference diagnosis is also determined by a consensus procedure among experts based on clinical information on the course of symptoms over a two-year time period and applying the core clinical criteria for the diagnosis of dementia due to AD [8] and core clinical criteria for the diagnosis of MCI due to AD [7]. Experts are kept blind for any information of the emerging markers under evaluation. Evaluating all cases by expert panel discussion meetings is highly time consuming. Therefore, first expert raters will assess all cases by means of an internet based form and if consensus is not reached the case will be discussed by a panel discussion meeting. The consensus diagnosis during the expert panel meetings is based on a modified Delphi method in which face-to-face discussions are held [45,46].

### Analyses

Several diagnostic procedures based on current practice and emerging biomarker information (index tests) are compared to the reference diagnosis. First, AD aetiology based on the core clinical criteria [7,8] is evaluated. This is reflected by the baseline diagnosis as determined by the consensus procedure. No information of any emerging biomarker is included. Second, AD aetiology based on the research criteria as established by the National institute on aging and the Alzheimer’s Association are evaluated [9]. At last, several explorative decision rules are applied including clinical information and biomarker information to determine AD aetiology and suspected progression of cognitive decline within two years.

#### Research goal 1: Diagnostic test accuracy

Diagnostic test accuracy is determined by relating index test results to the reference diagnosis (reference test). All diagnoses consist of a dichotomous outcome value on underlying pathology of the clinical syndrome and on (expected) progression of cognitive decline within two years. Separately for aetiology and progression of cognitive decline each index test result for each case is indicated as either true positive, true negative, false positive or false negative based on the reference diagnosis (reference test). This enables the calculation of accuracy estimates: sensitivity, specificity, positive predictive value, negative predictive value, likelihood ratio and (an increase of) the Area Under the Curve of a Receiver Operating Characteristic curve.

Finally, novel innovative diagnostic tests are developed during the course of this study by other cooperating researchers for which proof of principle does not yet exist. These tests will be evaluated as soon as evaluation in clinical subjects is possible. To assess diagnostic accuracy the tests will be applied in subgroups of the cohort retrospectively (for CSF samples) or using a case–control design.

#### Research goal 2: Cost-consequence and cost-effectiveness analysis

A cost-consequence analysis is performed listing all relevant costs and effects without aggregating it into a ratio [47] allowing decision-makers to choose the outcome of particular interest to include in an economic analysis. Average costs and consequences of the whole cohort are compared with the subgroup of patients who have received a correct diagnosis according to the reference test. This enables the comparison of the current diagnostic practice costs and effects and the costs and effects of an ideal situation (a costless diagnostic test with 100% accuracy). The difference indicates the maximum possible achievable benefit of new biomarkers for AD and related disorders in terms of costs and health effects. Furthermore, a cost-effectiveness analysis is performed comparing current practice with diagnostic procedures that include emerging biomarkers. The change in costs is compared to the change in diagnostic accuracy to obtain a cost-effectiveness ratio in terms of costs per correctly diagnosed patient.

#### Research goal 3: Decision analytic model

Finally a decision analytic model is built which provides a framework combining available evidence from different resources among which (accuracy) results from the study, literature and expert opinion. A decision analytic model can be defined as a set of mathematical relationships that form a structure reflecting the natural progression of a disease. By simulating patients or fractions of a population, these models enable the estimation of the likelihood of each consequence and its corresponding costs and effects [48]. It is applied to evaluate the short-term cost-effectiveness in terms of cost per correct diagnosis and long-term cost-utility in terms of cost per quality adjusted life year gained of diagnostic strategies under evaluation. Utility scores will be used to calculate Quality Adjusted Life Years (QALY). Patients who pass away during the year covered by the evaluation will be given a utility score of zero from the exact time of death. QALYs will be derived using the trapezium rule. A societal viewpoint will be adopted including the evaluation of all relevant costs and effects to calculate the societal benefits.

The cohort design facilitates the evaluation of many different diagnostic procedures by varying the place of a new diagnostic marker in the clinical pathway. Each procedure generates specific proportions of correct or incorrect diagnoses. Average costs and effects of correct and incorrect diagnoses are applied to calculate the total costs and effects of each procedure.

Sensitivity analysis will be performed taking into account both first order uncertainty regarding variation between patients in a homogeneous group and second order uncertainty regarding the true value of the parameters included in the model. This also enables the evaluation of an earlier diagnosis, different test sequences and the effect of possible new disease modifying drug treatments.

### Sample size and missing data

A telephone interview is performed for patients who refuse follow-up assessments to determine the reason for refusal, possible cognitive decline and interference with daily activities, and to assess the CDR. Incomplete data will be imputed by means of a regression model. Complete missing data or data missing covariates will be imputed using Rubin’s multiple imputation (MI) procedure.

Sample size is based on an 80% accuracy of current clinical practice to determine correct aetiology [4] and 70% in non-demented patients [49]. Applying a type I error (α = 5%), type II error (β = 80%), drop-out rate of 10% and minimum clinically relevant difference of 10% accuracy increase requires 219 patients to be included in the study.

Contrary to clinical studies, economic evaluations are not based on testing hypotheses. Their goal is to assess decision uncertainty. Therefore, economic evaluations are restricted to the estimation of the uncertainty surrounding cost-effectiveness (expressed in a statistical confidence interval). Within this Bayesian framework, classical inference (and therewith a power analysis) is irrelevant [50].

### Ethical considerations

According to the medical ethics committee “MedischEthischeCommissieazM/UM” the research protocol complies with the Declaration of Helsinki (October 2008, http://www.wma.net, ref.nr.: MEC 09-3-038) and with the Medical Research Involving Human Subjects Act and codes on ‘good use’ of clinical data and biological samples as developed by the Dutch Federation of Medical Scientific Societies.

## Discussion

This research protocol describes the methods used to assess the clinical and economic value of new diagnostic approaches for the diagnosis of AD. A delayed-type cross-sectional accuracy study design is chosen because a randomized clinical trial comes with ethical issues, long follow-up time and limited power. Two hundred forty one consecutive patients suspected of having a primary neurodegenerative disease are followed up for two years and a reference diagnosis is determined by an independent consensus expert panel. Eligibility criteria are chosen to maximally reflect a patient cohort within clinical practice.

Several other multi-centre trials study the relative value of new biomarkers for early evaluation of AD and related disorders. The Alzheimer’s Disease Neuroimaging Initiative (ADNI) in North America is aimed to identify neuroimaging measures and biomarkers associated with cognitive and functional changes in healthy elderly subjects and in subjects who have MCI and AD [51]. Furthermore, the ‘Development of Screening Guidelines and Clinical Criteria for Predementia AD’ (DESCRIPA) study is aimed to develop screening guidelines for predementia AD in the general population [52]. Both include markers in PET, MR imaging and CSF. The uniqueness of this study is the assessment of resource utilization and quality of life to enable an economic evaluation. Furthermore, the decision analytic model enables the evaluation of the optimal diagnostic strategy and the evaluation of diagnostic techniques to be developed during the study in sub-cohorts of the study population. At last, without a disease modifying treatment, the added value of biomarkers is uncertain. Therefore, the availability of such treatment is explored in the sensitivity analysis.

The study has some limitations. It focuses on applying new tests for diagnostic or prognostic goals. Screening and treatment monitoring are outside the scope of this study. A follow-up period of two years was taken as a compromise to maximise the time for the disease to express symptoms of progression (to prevent false negative reference diagnoses) and to minimize the time to prevent the start of a new disease episode after the baseline assessment (to prevent false positive reference diagnosis). It may take up to 10 years before all symptoms of dementia come to expression in subjects with AD pathology [53].

The study results are generalizable to a population of patients who are referred to a memory clinic of a university medical centre due to their memory problems.

## Competing interests

The author(s) declare that they have no competing interests.

## Authors’ contributions

CW designed the study and assisted RH in drafting the manuscript. FV (project leader) designed the study. JS (project leader) designed the study. MOR designed the study. PA (project coordinator) designed the study. PJV designed the study. PS designed the study. RH designed the study and drafted the manuscript. All authors read and approved the final manuscript.

## Pre-publication history

The pre-publication history for this paper can be accessed here:

http://www.biomedcentral.com/1471-2377/12/72/prepub
